# Comparative study of matrix metalloproteinase expression between African American and Caucasian Women

**DOI:** 10.1186/1477-3163-3-15

**Published:** 2004-10-29

**Authors:** Jacquline A Mason, Haile F Yancy, Kerrie Lashley, Marty Jett, Agnes A Day

**Affiliations:** 1 Department of Microbiology, College of Medicine Howard University, Washington, D.C. 20059, USA; 2 Department of Biology Howard University, Washington, D.C. 20059, USA; 3 Division of Pathology, Walter Reed Army Institute of Research, Washington, D.C. 20021, USA

## Abstract

To date there are 26 human matrix metalloproteinases (MMPs) which are classified according to their substrate specificity and structural similarities. The four major subgroups of MMPs are gelatinases, interstitial collagenases, stromelysins, and membrane-type matrix metalloproteinases (MT-MMPs). This study investigates the expression of 26 MMPs, which have been shown to play a role in cancer metastasis. Breast tissues and cell lines derived from African American patients and Caucasian patients were assayed to demonstrate alterations in the transcription of genes primarily responsible for degrading the extracellular matrix (ECM). The expression levels of the extracellular matrix and adhesion molecules were analyzed using the gene array technology. Steady state levels of mRNAs were validated by RT-PCR analysis. Total RNA was isolated from tissue and cell lines and used in the RT-PCR assays. From this data, differential expression of MMPs between 6 breast cancer cell lines and 2 non-cancer breast cell lines was demonstrated. We have performed an *in vitro *comparison of MMP expression and established differences in 12 MMPs (3, 7, 8, 9, 11–15, 23B, 26, and 28) expression between African American and Caucasian breast cell lines. Thus, evidence indicates that altered expression of MMPs may play a role in the aggressive phenotype seen in African American women.

## Introduction

In 2003, it was estimated that approximately 1.3 million Americans would be diagnosed with invasive cancer. Of this group, racial/ethnic minorities account for a disproportionate number of these cancers [1,2]. Invasive breast cancer usually begins in either the lobules or the ducts of the breast. These tumors then metastasize via the breast associated and thoracic lymphatic tissue [3]. The incidence of breast cancer in Caucasian women (112 out of 100,000) is higher than in African American women (AA) (95 out of 100,000) after the age of 40, however, the mortality suffered by (AA)(37 out of 100,000) is higher than Caucasian women (CAU) (31 out of 100,000) at every age [4]. Thus a greater percentage of AA women die from breast cancer and resulting metastasis. In 2004, an estimated 215,990 new cases of invasive breast cancer is expected to occur among women in the United States [5]. Breast cancer is the most common cancer among AA women; however, the rate of newly diagnosed cases is about 13% lower than CAU women [6]. There is accumulating evidence that AA women have a higher frequency of more aggressive tumor types, which have been shown to lead to higher mortality rates. Studies show that compared Caucasian women (CAU), African American women (AA), regardless of age had proportionally more Grade III tumors and fewer Grade I and II tumors for all stages combined and for each individual stage group [7]. The grade of cancer has been shown to be a prognostic factor with higher-grade tumors being associated with reduced survival [7]. The most common cause of death in breast cancer patients is metastasis of breast cancer cells to bones, lungs, liver and brain and the progressive growth of the cancer at these sites [7,8]. Therefore, controlling breast cancer metastasis represents an effective method of preventing or slowing disease progression.

The extracellular matrix (ECM) is a complex structural entity surrounding and supporting organs and tissues of the body. The ECM plays a key role in cell-cell signaling, wound repair, cell adhesion and tissue function. Recent studies suggest that cell adhesion proteins located on breast cancer cells interact with the ECM [7]. This interaction induces increased production by the breast cancer cells of proteins that degrade the ECM. This degradation enables the tumor cells to invade the surrounding tissue and ultimately enter the circulatory system. Once they are in circulation, tumor cells travel to other organ sites where they progressively grow [7].

The matrix metalloproteinases (MMPs) are a family of structurally and functionally related endoproteinases that are involved in the degradation of the ECM. Currently, there are 26 identified human matrix metalloproteinases, which are classified according to their substrate specificity and structural similarities [8]. Abnormal expression of these proteins contributes to various pathological processes including rheumatoid arthritis and tumor growth, invasion and metastasis. The four main subgroups of MMPs are the interstitial collagenases, which catalyze degradation of fibrillar forms of collagen, the gelatinases which degrade gelatin and collagen that are abundant in basement membranes, the stromelysins, which degrade various substrates including proteoglycans, laminin and collagen I, II, and III and the membrane-type MMPs which have been shown to catalyze activation of progelatinase A, to degrade a variety of ECM substrates and to function as a fibrinolytic enzyme in the absence of plasmin [9].

MMP expression has bee shown to be elevated during development, pregnancy, and involution and has been shown to be related to tumor cell invasiveness [10]. This study investigates the expression levels of the 26 identified MMPs, which have been shown to play a role in the metastatic process using breast tissues and cell lines derived from AA and CAU women.

## Materials and Methods

### Cell Culture and Tissue RNA

All cell lines were purchased from American Type Culture Collection (Rockville, MD, USA). Cells were propagated in the recommended media and given new media every 2 to 3 days until 90% confluent (see table 1). Human Breast Tissue RNA was purchased from Ambion (Austin, TX).

### RNA Extractions

RNA was extracted from the cell line using the RNAqueous (Ambion, Austin, TX). Cells were collected by low speed centrifugation and lysed by adding 200 μl of Lysis/Binding Solution. An equal volume of 64% ethanol was added to the lysate. The lysate/ethanol mixture was transferred to the RNAqueous Filter Cartridge and centrifuged for 1 minute at 13,400 rpm. The flow through was discarded and 700 μl of Wash Solution 1 was added to the RNAqueous Filter Cartridge and centrifuged for 1 minute. The column was washed twice with 500 μl of Wash Solution 2/3 and eluted with 110 μl Elution Solution. Isolated RNA was quantitated using the UltraSpec 2000 (Pharmacia Biotech). All RNA samples were stored at -70°C in RNA elution solution until further use.

### Gene array

The Extracellular Matrix and Adhesion Molecule gene arrays were obtained from SuperArray (Frederick, MD). The array membranes were pre-hybridized with GEA hybridization solution and denatured salmon sperm DNA at 60°C for two hours. For each RNA sample, a labeling mix consisting of 4 μl 5X GEA labeling buffer, 2 μl biotin-16-dUTP, 1 μl RNase inhibitor, 1 μl reverse transcriptase, and 2 μl RNase-free water was prepared and an aliquot of 3 μg of RNA was added to each respective thin-walled PCR tube. The cDNA labels were created using a cycle of 3 minutes at 70°C, 2 minutes at 42°C, and an additional 90 minutes at 42°C. Two microliters of stop buffer was added and the mix denatured at 94°C for 5 minutes. The labeled cDNA was added to the membrane and allowed to hybridize overnight. The membranes were washed with 2X SSC/0.1% SDS and 0.1X SSC/0.5% SDS, blocked with blocking solution, and the probes were detected using AP-Strepavidin, specific buffers, CDP-Star and subsequent exposure to X-ray film for 30 seconds to 5 minutes. The autoradiograms were analyzed using ScanAlyzer and GEArray Analyzer (SuperArray, Frederick, MD).

### Reverse Transcriptase Polymerase Chain Reaction (RT-PCR)

The RT-PCR reactions were performed in a P/E GeneAmp 9700 thermocycler (Perkin-Elmer Co., Norwalk CT), using the Access RT-PCR system (Promega, Madison, WI). The reaction mixes were prepared by combining 27. 5 μl of nuclease free water, 10 μl of AMV, 1 μl Tfl 5X reaction buffer, 2 μl dNTP mix, 50 pM of upstream primer, 50 pM of downstream primer in 1.5 μl volume each, 3 μl 25 mM MgSO_4_, 1.0 μl AMV reverse transcriptase, Tfl DNA polymerase and 1 μg of total RNA in a 0.5 ml thin walled Eppendorf tube on ice. The reaction mixes were then vortexed for 5 seconds and centrifuged. The PCR cycling profile was as follows: 48°C for 1 minute for reverse transcription of the RNA into cDNA, 94°C for 4 minutes to deactivate the reverse transcriptase, and 30 cycling sequences of denaturing at 94°C for 45 seconds, annealing at 55°C for 30 seconds, and extension at 72°C for 1 minute with a final extension at 72°C for 10 minutes. An aliquot of 20 μl of each RT-PCR reaction were run on 1.2% agarose gels, stained with ethidium bromide, photographed and subjected to densitometic measurements using the Chemi-Imager Tm 4000 (Alpha Innotech, Corporation, San Leandro, CA).

## Results

### Gene Array Analysis

Gene arrays were utilized to explore and compare the expression levels of extracellular matrix adhesion molecules in AA and CAU breast cancer cells. The array revealed elevated expression in 36% of the genes in AA samples when compared to their CAU counterparts. Of those elevated genes, 31% were from the cell membrane adhesion molecules group, 17% from the extracellular matrix proteins functional gene group, 37% from the proteases category, and 14% were protease inhibitors. Initial results of the gene array indicated a significant elevation of the proteases (data not shown). To further evaluate this, we proceeded with direct analysis of all known MMPs.

### MMP RT-PCR Analysis

**Figure 1:**
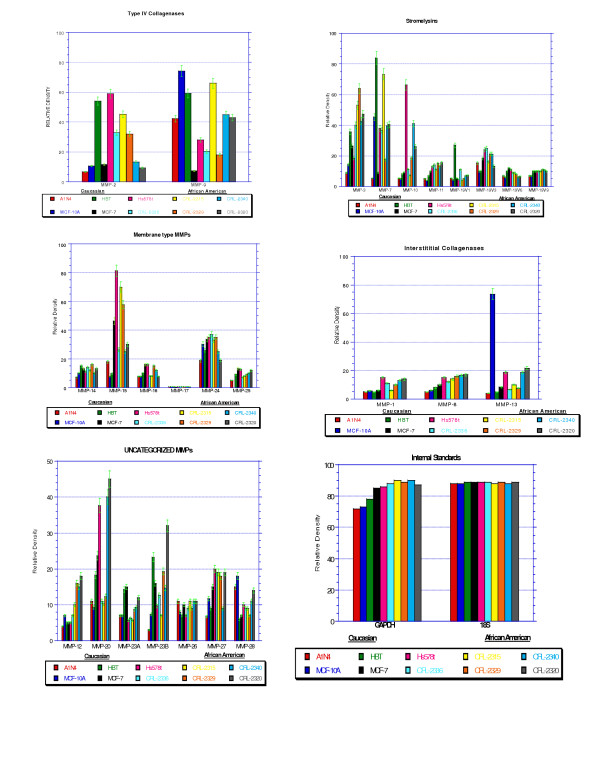
RT-PCR expression of MMPs in African American and Caucasian breast cell lines and tissue.

Comparison of the individual relative densities between AA and CAU women revealed elevated expression in 12 of the 26 MMPs (3, 7, 8, 9, 11, 12, 13, 14, 15, 23B, 26, and 28) (Figure 1 and Table 2). Elevated expression of MMP-3, 7, 8, 9, 11, 12, 13, 14, 23B, 26 and 28 in AA breast cancer cells was observed when the overall averages of the expression levels of all AA and CAU women cell lines were compared (Table 3).

**Table 2:** Matrix Metalloproteinase Expression Assessment by RT-PCR

		**African American**			**Normal**		**Caucasian**		
	**Cell Lines**	**2315**	**2320**	**2329**	**A1N4**	**10A**	**MCF7**	**Hs578t**	**2336**

**MMPs**									

1		6 ± 2.7	12 ± .58	11 ± 1.15	5	5.7	6 ± 2.7	15 ± 2	11 ± 2.1
2		45.3 ± 4.2	32 ± .58	13.3 ± .58	6.7	11	11.7 ± 7.8	59 ± 1	33 ± 11
**3**		**53 ± 11.5**	**47.3 ± 11**	**64 ± 7**	**9**	**14.3**	**26.5**	**18.3**	**40**
**7**		**73.3 ± 2.3**	**40.67 ± 2.1**	**17.6 ± 2.5**	**5.3**	**45.3**	**9 ± 1.7**	**38 ± 14.53**	**36.7 ± 4**
**8**		**14 ± 2.5**	**17 ± 2.5**	**16 ± 2.1**	**5**	**6**	**10 ± 1**	**15 ± 1.2**	**12 ± 1**
**9**		**66 ± 3**	**43 ± 5.2**	**18 ± 5.5**	**42.3**	**74.3**	**7.3 ± 1.5**	**28 ± 2.7**	**20.3 ± 5.13**
10		7.3 ± .58	26 ± 1.7	18.7 ± 5.5	5.3	5	9.3 ± 1.15	66.3 ± 4	11.3 ± 1.5
**11**		**11 ± 2**	**15.7 ± 2.9**	**15 ± 5**	**5**	**4**	**10 ± 4**	**13 ± 3.79**	**14 ± 0.58**
**12**		**10 ± 4**	**18 ± 8.9**	**16 ± 3.6**	**4**	**7**	**5 ± 1.15**	**5 ± 1**	**7 ± 2.1**
**13**		**10 ± 2**	**21.7 ± 4.2**	**7.7 ± 1.2**	**4**	**73.67**	**8.3 ± 2.1**	**18.7 ± 3.5**	**6.7 ± 1.15**
**14**		**12 ± 3**	**13.7 ± 1.5**	**16 ± 5**	**7**	**10**	**13 ± 8.5**	**11 ± 1.5**	**14 ± 3.1**
**15**		**70 ± 2.7**	**30 ± 0.58**	**57.7 ± 6.3**	**18**	**7.67**	**46.3 ± 4.5**	**81.3 ± 7.5**	**26.3 ± 6.8**
16		8.7 ± 4	7.7 ± 1	15.7 ± 4	7.67	7.67	16 ± 2.9	16 ± 1	8 ± 2.7
17		N/D	N/D	N/D	N/D	N/D	N/D	N/D	N/D
19v1		4 ± 1	7.3 ± .58	5 ± 1	5.33	4.33	5.3 ± 1.5	4.7 ± .58	11 ± 3.5
19v3		16 ± 6	13.3 ± 2.9	21.3 ± 8.7	15.3	10	18.3 ± 6.1	24 ± 4.2	25 ± 5
19v6		9.7 ± 4.6	9 ± .58	8 ± 5.2	7	6	12 ± 2.1	11 ± 2.1	9 ± 2.7
19v9		10 ± 1.0	10 ± 3.5	11 ± 2.0	7	7	10 ± 5	10 ± 2.0	10 ± 2.3
20		10.3 ± 3.2	45 ± .58	12.3 ± 2.1	11	9.3	23.7 ± 12.4	37.7 ± 6.0	11 ± 1
23A		5.7 ± 1.5	12 ± 2.0	8.7 ± 1.5	7	7	10 ± 2.8	5.7 ± .58	6.3 ± .58
**23B**		**7 ± 1.1**	**32 ± 10.6**	**19.3 ± 1.5**	**3**	**7.3**	**10 ± 1.4**	**9.7 ± 1.5**	**12.7 ± 3.2**
24		33 ± 1.2	19.7 ± 1.2	36 ± 1	18.67	30.2	33.7 ± 1	35 ± .58	37 ± 1.5
25		8 ± 1	12 ± 5.2	9 ± 1.0	5	6	13 ± 1.5	12.7 ± 1.0	7 ± 2.0
**26**		**11 ± 1.0**	**11 ± 1.7**	**9 ± 2.5**	**11**	**8**	**10 ± 2**	**7 ± .58**	**9 ± 1.0**
27		19 ± 7.2	19 ± 6.0	18 ± 8.0	6.7	11.67	15 ± 2.0	20 ± 13.2	19 ± 1.0
**28**		**9 ± 5.6**	**14 ± 5.7**	**7 ± 2.0**	**15**	**18**	**7 ± .58**	**10 ± .58**	**9 ± 6.0**

**RT-PCR expression of MMPs in AA and CAU cells. Elevated expression in AA vs. CAU denoted in bold. Mean ± SD **N/D: not detected

**Table 3:** Averaged Relative Density of MMP Expression For AAW CAU and Normal Cell Lines

	**AAW**	**CAU**	**Normal**
**MMPs**			
1	3.2	3.4	5.35
2	9.6	11.5	11.3
**3**	**18.3**	**8.4**	11.6
**7**	**14.63**	**9.3**	25.3
**8**	**5.37**	**4.18**	5.5
**9**	**14.1**	**6.2**	58.3
10	5.8	9.7	5.1
**11**	**4.62**	**4.2**	4.5
**12**	**4.9**	**2**	5.5
**13**	**4.4**	**3.7**	38.8
**14**	**4.63**	**4.3**	8.5
15	17.5	17.1	12.8
16	3.6	4.5	7.67
17	N/D	N/D	N/D
19v1	1.8	2.3	4.83
19v3	5.6	7.5	12.65
19v6	3.0	3.6	6.5
19v9	3.4	3.4	7
20	7.5	8.0	10.1
23A	2.9	2.4	7
**23B**	**6.5**	**3.7**	5.1
24	9.9	11.7	24.4
25	3.2	3.7	5.5
**26**	**3.5**	**2.9**	9.5
27	6.1	6	9.1
**28**	**3.3**	**2.96**	16.5

**RT-PCR expression of MMPs in AA, CAU cancer cell lines and Normal cell lines.. Elevated expression in AA -vs- CAU denoted in bold. **N/D: not detected

## Discussion

Little is known as to why the incidence of breast cancer is lower yet mortality is higher in African American women. Many studies speculate that this is only a socio-economical problem [11]. However this investigation provides another possibility that may reveal molecular mechanisms that contribute to the increased mortality of AA women with breast cancer. The major threat to patients with breast cancer is tumor invasion and metastasis [12]. Tumor invasion is a complex process that requires interaction between the invasive cells and the ECM [13]. This process involves a cascade of events including angiogenesis, local invasion, and intravasation. One of these critical steps involves the proteolytic degradation of the ECM and basement membrane. This is partially done by the matrix metalloproteinases. One aspect related to cancer progression has been considered in numerous studies is the association of MMP expression with tumor grade and aggressiveness [14].

The GEArray Q Series Human Extracellular Matrix and Adhesion Molecules Gene Array were used to determine the expression profiles of various types of matrix and adhesion molecules. The array was divided into four components: cell adhesion molecules, extracellular matrix proteins, proteases and protease inhibitors. From analysis of the gene array, altered expression was observed in many of the proteases. These findings led to further study of the matrix metalloproteinases (data not shown).

Gene Array analysis of AA and CAU breast cancer cells indicates that there is altered expression of the genes in the Extracellular matrix and adhesion molecules, particularly the proteases. This group included 17 MMPs of which ten displayed elevated expression in AA women.

RT-PCR was performed to confirm the results of the gene array. We observed elevated expression of 12 MMPs in AA cell lines when compared to their CAU counterparts. These include one gelatinase (MMP-9), two interstitial collagenases (MMP-8, and 13), 3 stromelysins (MMP-3, 7, 11), two MT-MMPs (MMP-14 and -15) and 4 uncategorized MMPs (MMP-12, 23B, 26, and 28). There was no MMP-17 expression detected in any of the cell lines (Figure 1).

Studies have shown that normal mammary gland expression of MMPs is low except during times of development, pregnancy, and involution [10,15,16]. However, during pathologic states such as breast cancer, increased levels of MMPs have been reported in breast tumor cells as well as in the surrounding non-cancerous breast tissue [17].

Our results suggest that there is altered expression of MMPs in cell lines derived from AA and CAU women. It also demonstrates that there is greater expression of MMPs in AA women than in CAU women. This investigation indicates that altered expression of MMPs may play a role in the aggressive phenotype seen in AA women. This evidence suggests that the elevated expression levels of 12 MMPs may be a contributing factor in the higher mortality rates of AA breast cancer patients. This study is significant because it may reveal biomarkers of metastasis in AA women. To date, this is the first study to extremely investigate MMP expression in cell lines derived from African American patients.

## Abbreviations used

MMP-Matrix metalloproteinases, MT-MMP-Membrane-type matrix metalloproteinases, AA-African American women, CAU-Caucasian, RT-PCR-Reverse transcriptase Polymerase Chain Reaction, ECM-Extracellular matrix

## Author's Contributions

JAM performed the microarrays and RT-PCR experiments, was involved in tissue culture and prepared the first draft of the manuscript. HFY was responsible for primer design, and performed data analysis and densitometric readings of the gene arrays and RT-PCR. KL maintained all cells and tissues and assisted in the editing of this manuscript. MJ provided cell lines, training in microarray performance and editing. AAD conceived the study and participated in its design, coordination and funding, as well as preparation of the manuscript. All authors read and approved the final manuscript.

**Table 1:** Cell Lines and Tissue Samples

Human Breast Tissue	Normal breast tissue (derived from CAU)
MCF-10A	Mammary gland, fibrocystic disease (CAU)
A1N4	Mammary epithelial, chemically transformed (CAU)
	**CAUCASIAN (CAU)**
HS578T	Mammary gland; breast; carcinoma
MCF-7	Mammary gland; breast; epithelial; metastatic site: pleural effusion adenocarcinoma
CRL-2336	Mammary gland epithelial, primary ductal carcinoma
	**AFRICAN AMERICAN (AA)**
CRL-2315	Breast, primary ductal carcinoma
CRL-2329	Carcinoma, ductal, primary; breast; mammary gland
CRL-2320	Carcinoma, ductal, breast; mammary gland; from metastatic site: lymph node
